# Effect of Continuous Touch on Brain Functional Connectivity Is Modified by the Operator’s Tactile Attention

**DOI:** 10.3389/fnhum.2017.00368

**Published:** 2017-07-20

**Authors:** Francesco Cerritelli, Piero Chiacchiaretta, Francesco Gambi, Antonio Ferretti

**Affiliations:** ^1^Department of Neuroscience, Imaging and Clinical Sciences, “G. D’Annunzio” University of Chieti-Pescara Chieti, Italy; ^2^ITAB-Institute for Advanced Biomedical Technologies, “G. D’Annunzio” University of Chieti-Pescara Chieti, Italy; ^3^Clinical-Based Human Research Department—C.O.M.E. Collaboration ONLUS Pescara, Italy

**Keywords:** affective touch, osteopathic manipulative treatment, tactile stimuli, fMRI, insula

## Abstract

Touch has been always regarded as a powerful communication channel playing a key role in governing our emotional wellbeing and possibly perception of self. Several studies demonstrated that the stimulation of C-tactile afferent fibers, essential neuroanatomical elements of affective touch, activates specific brain areas and the activation pattern is influenced by subject’s attention. However, no research has investigated how the cognitive status of who is administering the touch produces changes in brain functional connectivity of touched subjects. In this functional magnetic resonance imaging (fMRI) study, we investigated brain connectivity while subjects were receiving a static touch by an operator engaged in either a tactile attention or auditory attention task. This randomized-controlled single-blinded study enrolled 40 healthy right-handed adults and randomly assigned to either the operator tactile attention (OTA) or the operator auditory attention (OAA) group. During the five fMRI resting-state runs, the touch was delivered while the operator focused his attention either: (i) on the tactile perception from his hands (OTA group); or (ii) on a repeated auditory stimulus (OAA group). Functional connectivity analysis revealed that prolonged sustained static touch applied by an operator engaged with focused tactile attention produced a significant increase of anticorrelation between posterior cingulate cortex (PCC-seed) and right insula (INS) as well as right inferior-frontal gyrus but these functional connectivity changes are markedly different only after 15 min of touching across the OTA and OAA conditions. Interestingly, data also showed anticorrelation between PCC and left INS with a distinct pattern over time. Indeed, the PCC-left INS anticorrelation is showed to start and end earlier compared to that of PCC-right INS. Taken together, the results of this study showed that if a particular cognitive status of the operator is sustained over time, it is able to elicit significant effects on the subjects’ functional connectivity patterns involving cortical areas processing the interoceptive and attentional value of touch.

## Introduction

Touch is a critical communication channel across lifespan. The sense of touch is divided into two major categories: proprioceptive and interoceptive (affective), activated by distinct mechanisms with cerebral correlates in somatosensory and insular cortex, respectively (Olausson et al., 2002; McGlone et al., 2012).

While the proprioceptive aspects of touch were largely studied from both neurophysiological and neuroscience standpoints, the interoceptive properties were only recently considered and hypothesized as crucial for social interaction (Terasawa et al., 2013), empathy (Ernst et al., 2013) and eventually touch based treatments (McGlone et al., 2017).

Neuroimaging studies in neuronopathy patients who have lost all “fast” 1st touch nerves, and healthy controls, showed that gentle stroking touch (later referred also as affective touch, see McGlone et al., 2014) applied to hairy skin, but not palmar skin, reliably produces activation in the insular (interoceptive cortex) and orbitofrontal cortex (reward) as opposed to primary somatosensory cortex (Olausson et al., 2002; McGlone et al., 2012). This affective touch was demonstrated to be mediated by unmyelinated “slow” mechanosensitive nerves in the skin (called C-Tactile afferents—CTs), which respond optimally to low velocity, low force stroking movements such as gentle brushing (Vallbo et al., 1999; McGlone et al., 2014), but are also temperature sensitive (Ackerley et al., 2014) and triggered by static touch (Lindgren et al., 2012).

Several brain studies further confirmed these preliminary findings (Essick et al., 1999, 2010; Loken et al., 2009; Fairhurst et al., 2014), using different touch-based modalities, highlighting the role of CTs in the central representation of the physical condition of the body (Craig, 2002, 2009; Björnsdotter et al., 2010). Finally, McGlone et al. (2017) recently hypothesized a CT-based affective homunculus within the insular cortex (McGlone et al., 2017).

A crucial aspect in the context of brain processing of touch, in particular affective touch, is the interaction with different types of subject attention. As a matter of fact, the association between touch and attention (overt, covert, endogenous or exogenous) is considered important for processing and interpreting the different peripheral stimuli (for review see Spence, 2002). Different tactile attention tasks performed by subjects receiving the touching seem to alter the perception of touch and its brain representation, with observed effects in the default mode network (DMN) and its anticorrelated areas such as the insular cortex (Gallace and Spence, 2014).

Touch has been always regarded as a powerful communication channel (Gallace and Spence, 2010) playing a key role in governing our emotional wellbeing (Field, 2014) and possibly perception of self, i.e., interoceptive reactions. A particular aspect of touch that has been poorly investigated is the role of who gives the touch. It is notable here that interpersonal tactile communication is a bi-directional process, therefore potentially involving a cognitive modulation by both subjects. Indeed, studying interpersonal touch taking into account the cognitive and neural correlates of tactile perception of both subject and operator constitutes an important issue at present (see Gallace and Spence, 2008, 2009). Indeed, whether we receive a pleasant caress, a massage, a pat on the back, a manual treatment, a handshake, or a gentle brush of the shoulder, our experience seems to suggest the ability to perceive the emotional and cognitive state of who is giving the touch. Existing studies, however, were only conducted on the top–down attentional modulation (Rolls, 2008) of touch by who receive the tactile stimulation, showing activation of the orbitofrontal and cingulate cortexes (McCabe et al., 2008) as well as SII.

Nevertheless there is no evidence on whether different attention states of the person/operator performing the touching would produce different brain responses on subjects being touched. In this study we investigated functional connectivity while subjects were receiving a static touch by an operator engaged in either a tactile attention or auditory attention task. In particular we compared the effect of the two attention states of the operator on subjects brain connectivity, investigating DMN and its anticorrelated areas.

## Materials and Methods

This randomized-controlled single-blinded study enrolled 40 healthy right-handed subjects, of either gender, aged between 18 and 30 years old, and who did not undertake any pharmacological treatment during the previous 4 weeks. Exclusion criteria included: any cardiovascular, neurological, muscle-skeletal, psychiatry, genetic or congenital disorders, any contraindication to MRI scanning, including metal implants and claustrophobia and current pregnancy or breastfeeding. Smokers as well as drug abuse subjects were excluded. Participants were asked to refrain from alcohol, caffeine and cardiovascular exercise for 24 h prior to the experimental session to control for external confounders.

The Institutional Ethics Committee of the University “G. D’Annunzio” of Chieti-Pescara approved the study and written informed consent from all subjects were obtained before the experiment according to the Declaration of Helsinki.

## Randomization

Subjects underwent an MR imaging protocol and were randomly divided into two groups using a 1:1 ratio and were assigned to either the operator tactile attention (OTA) or the operator auditory attention (OAA) group. Block randomization was performed according to a computer-generated randomization list using a block size of 10. Subjects were unaware of the study design and outcomes as well as of group allocation. The randomization was performed and stored in a secure web-based space and an information technology consultant was responsible for the process.

## Prescan Behavioral Assessment

Before the functional magnetic resonance imaging (fMRI) scan, subjects were asked to complete paper-based questionnaires. The socio-demographic questionnaire was administered to collect baseline data in terms of age, gender, BMI, civil state, academic degree, type of work and smoking habits. The State-Trait Anxiety Inventory (STAI-Y1 and Y2) was used to test trait anxiety (Spielberger et al., 1983) and the Edimburgh Handedness inventory was utilized to investigate the hand dominance (Oldfield, 1971).

## Experimental Design: Description of The Touch and Attention Task Protocol

The experiment consisted of five fMRI runs, each lasting 5.5 min. During the first run (baseline), subjects received no touch. In the remaining four runs (study period), a male experimenter/operator was standing aside the scanner bed and applied with hands a bilateral constant static light skin-to-skin pressure in proximity of the subject’s external malleolus. The level of the applied force (0.2 N) was chosen from the literature where Loken et al. (2009) reported that CT-fibers stimulated with a brush showed maximal sensitivity for movements characterized by a normal force on the skin of 0.2–0.4 N. Since no differences between brush and human touch were shown, the established normal force of 0.2 N was selected to produce a similar physical stimulation to published studies. The operator underwent a training phase outside the scanner, using a device consisting of two semi-cylindrically shaped mock-ups, with force transducers to measure tangential and normal forces applied to the surfaces (Lindgren et al., 2012). During training, the operator had visual feedback of the applied normal force (set value = 0.20 N). A training period of 15 min allowed the operator to apply the nominal force value (0.2 N) without visual feedback with a sufficient precision, as tested in a control session, where the mean forces were 0.19 N (SD = 0.2) and 0.18 N (SD = 0.3) for the left and right hand, respectively (correlation coefficient: 0.92).

During fMRI, the touch was delivered while the operator focused his attention either: (i) on the tactile perception from his hands (OTA group); or (ii) on a repeated auditory stimulus (OAA group).

The focused tactile attention task performed by the operator consisted on voluntary diverting his attention towards the feeling/perception from the hands, i.e., the operator had to feel the tissue in terms of consistency, density, temperature, responsiveness and motility (e.g., myofascial movements).

The focused auditory attention task consisted in directing the operator’s attention towards acoustic stimuli (beeps) delivered through headphones. The beeps were delivered at a random interval included between 0.5 s and 2.0 s. The operator had to count the number of beeps per run.

In both tasks, the planned attention selection process was endogenous, that is voluntarily directing the attention to a particular event either tactile or auditory, and covert oriented, that is internally shifting the attention towards the stimulus. In addition, the position of the operator was kept equal across subjects, groups and runs. The attention tasks needed to be sustained for the entire period of contact with the subject, i.e., from run 2 to 5.

Subjects were asked to lie still and keep their eyes closed during acquisition. Foam padding was employed to minimize involuntary head movement. Furthermore, subjects were questioned after each run about the physical and subjective features of touch (mean pressure perceived, type and nature of touch). The number was reported via a visual analog scale adopted to quantify the level of touch pleasantness (0 = very unpleasant, 10 = very pleasant). Here, participants were provided with an MRI-compatible button pad in the right hand and instructed about which button to press to indicate their judgments about the type of touch felt. The scale was projected via an LCD projector onto a screen visible through a mirror mounted on the headcoil. The operator was blind to these subjects answers in order to avoid a conditioning effect that could have influenced the touch of the following run.

## fMRI Data Acquisition

Images were acquired with a Philips Achieva 3 Tesla scanner (Philips Medical Systems, Best, Netherlands) using a whole-body radiofrequency coil for signal excitation and an 8-channel phased-array head coil for signal reception. A high resolution structural volume was first acquired using a 3D fast field echo T1-weighted sequence (sagittal, matrix 256 × 256, FOV = 256 mm, slice thickness = 1 mm, no gap, in-plane voxel size = 1 × 1 mm, flip angle = 12°, TR = 9.7 ms and TE = 4 ms). Then, Blood Oxygen Level Dependent (BOLD) fMRI data were obtained using a gradient-echo T2*-weighted echo-planar (EPI) sequence with the following parameters: matrix 80 × 80, voxel size 3 mm × 3 mm × 3.5 mm, SENSE 1.8, TE = 30 ms, TR = 1.8 s, 185 volumes per run.

During fMRI, cardiac (ppu) and respiratory (belt) data were also acquired. Physiological signals were recorded using a pulse oximeter placed on a finger of the left hand and a pneumatic belt strapped around the upper abdomen. Cardiac and respiratory data were both sampled at 100 Hz and stored by the scanner’s software in a file for each run.

## Postscan Ratings

Several measurements were used at the end of the MRI session in order to assess the quality of received touch. The Touch Perception Task (Guest et al., 2011) was used to describe the type of touch perceived by the subjects during the scans. In addition, a 5-point Likert scale was administered to classify the touch received by subjects (1 = very light, 2 = light, 3 = moderate, 4 = heavy, 5 = very heavy).

In addition, the Amsterdam Resting State Questionnaire to report perception of own feeling during the scan (Diaz et al., 2013) was administered.

## Behavioral Data Analysis

Arithmetic mean and standard deviation as well as median, percentage and range were used to report the general characteristics of the study population. To compare the OTA group and OAA group at enrollment, univariate statistical tests, student *t* test and chi square test were performed. To study the independent effect of attention focused touch on primary and secondary endpoints, a repeated measure analysis based on linear mixed effect model was applied considering group differences (OTA vs. OAA) across time (baseline vs. experimental runs). To indicate statistical difference, two-tailed *P* values of less than 0.05 was considered. The significance threshold was further adjusted for multiple comparisons using Bonferroni’s correction. This data analysis was carried out using the R statistical program (v. 3.5.2).

## fMRI Data Preprocessing

Analysis of fMRI data was performed using AFNI^1^. The first five volumes of each functional run were discarded to allow T1 equilibration of the MR signal. The first preprocessing steps included despiking (AFNI’s “3dDespike”) to remove transient signal spikes from the EPI time series, RETROICOR (Glover et al., 2000) to remove signal fluctuations related to cardiac and respiratory cycles, slice scan time correction and motion correction. Motion correction was done by rigid body registration of EPI images to the sixth volume of the first run. Then, additional preprocessing was performed using ANATICOR (Jo et al., 2010) to remove further physiological and hardware related confounds. Briefly, a global nuisance regressor was obtained extracting the EPI average time course within the ventricle mask and local nuisance regressors were obtained calculating for each gray matter voxel the average signal time course for all white matter voxels within a 3 cm radius (Jo et al., 2010). These nuisance regressors and the six regressors derived from motion parameters were removed from the EPI timeseries of each run using AFNI’s @ANATICOR. Individual masks of large ventricles and white matter used in this approach were obtained from the structural scans segmentation using FreeSurfer^2^ and coregistered to EPI using an affine transformation.

Finally, preprocessed functional scans were normalized to the MNI space, spatial smoothed (6 mm FWHM) and band-pass filtered (0.01–0.1 Hz).

The framewise displacement (FD) and the root mean square value of the differentiated BOLD timeseries (DVARS) within a whole brain spatial mask were also calculated and used as quality control measures to inspect between-groups differences of motion effects potentially not accounted for by spatial registration and regression of motion parameters (Power et al., 2012, 2014).

## Functional Connectivity Analysis

First, seed-based resting state connectivity maps were created for individual subjects calculating the Pearson correlation coefficient (r-value) between the Posterior Cingulate Cortex (PCC) time series and the time series at each voxel. The PCC time series was derived averaging the time courses of voxels inside a sphere with 6 mm radius (Table 1). Individual correlation maps were converted using the z-Fisher transformation (z = atanh (r), where r is the correlation coefficient) to approach a normal distribution before calculating the random effect group analysis. A one-sample *t*-test was performed on the z-Fisher maps to obtain group statistical functional connectivity maps, separately for the five runs of the OTA and OAA groups. These group statistical maps were thresholded at *p* < 0.05 corrected for multiple comparisons using False Discovery Rate (FDR) and used to visually inspect the level of connectivity during the five runs for the two groups.

**Table 1:** ROI coordinates taken from the articles cited in methods.

ROI	*X*	*Y*	*Z*
Right insula	38	−3	9
Left insula	−42	−3	3
Right inferior parietal lobe	65	38	40
Left inferior parietal lobe	−63	38	45
Right inferior frontal gyrus	41	−24	9
Right dorsolateral prefrontal cortex	23	−33	48
Left dorsomedial prefrontal cortex	−6	−57	−2
Right angular gyrus	48	65	33
Left angular gyrus	−56	65	27

Then, to quantify statistically significant differences across groups and time, a number of spherical nodes (6 mm of radius) for regions known to be correlated and anticorrelated with PCC were defined using independent coordinates from the literature (Uddin et al., 2009; see Table 1) in order to avoid circularity problems in the analysis (Kriegeskorte et al., 2009).

Individual connectivity values were extracted from these regions of interest (ROI) and compared across groups and conditions using a repeated measure analysis based on multivariate modeling (MVM) approach as implemented in R (Chen et al., 2014).

Data were analyzed with a linear mixed effects model in R^3^, which estimates parameters using Maximum Likelihood Estimation and estimates effects using specific contrast matrices. The fixed factors were defined as the group (OTA vs. OAA) and time (baseline vs. experimental runs), and subject was entered as a random factor. Considering the nine ROIs taken into account, the number of statistical tests performed were 18. To guard against Type I error, contrasts were both assessed at *p* < 0.05 corrected for Bonferroni multiple comparisons.

## Results

### Prescan Behavioral Results

There were no significant differences in terms of age, gender, BMI and all other clinical, demographic, neuropsychological and behavioral parameters between the two groups (*P* > 0.10; Table 2).

**Table 2:** Description of the general characteristics of the sample in the operator tactile attention (OTA) and operator auditory attention (OAA) group.

	Group OTA (*N* = 20)	Group OAA (*N* = 20)	|t| > 0
Age	27.0 (5.4)	26.9 (4.1)	0.95
Male*	13 (65)	10 (50)	0.52
BMI	23.9 (3.7)	23.2 (2.9)	0.52
Marital status*			0.49
not married	17 (85)	19 (95)	
married	2 (10)	1 (5)	
divorced	1 (5)		
Education title*			0.35
secondary school	1 (5)	1 (5)	
high school	12 (60)	8 (40)	
academic degree	7 (35)	11 (55)	
Working condition*			0.13
student	8 (40)	13 (65)	
employed	11 (55)	5 (25)	
unemployed	0 (0)	2 (10)	
other	1 (5)	0 (0)	
STAI-Y1	45.3 (2.4)	45.4 (3.9)	0.90
STAI-Y2	44.8 (2.6)	46.1 (3.1)	0.21

Data are presented as mean (sd) and *N (%). P values from student *t* test or * chi square test.

### Postscan Ratings Results

No imbalances were found in terms of self-reported touch characteristics across groups (Table 3). Indeed, overall participants rated the touch as pleasant (according to TPT). For example, participants described the touch as pleasant, light, soft, comfortable, relaxing and cozy on the skin.

**Table 3:** Description of touch perception in the OTA and OAA group.

	Group OTA (*N* = 20)	Group OAA (*N* = 20)	|X| > 0
Touch rate	9 (5–10)	8 (6–10)	0.12
Type of touch*			0.80
very light	9 (45)	7 (35)	
light	10 (50)	12 (60)	
moderate	1 (5)	1 (5)	

Touch rate represents the scores obtained during the experiment. Data are presented as median (min-max) and *N (%). P values from Mann-Whitney test or * chi square test.

### Whole Brain Results

Table 2. Results of the experimental conditions.

**Figure 1:**
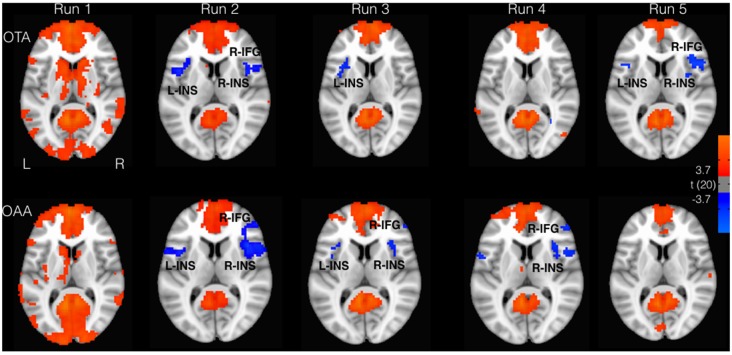
Areas correlated (red) and anticorrelated (blue) with posterior cingulate cortex (PCC; *p* < 0.05, false discovery rate (FDR) corrected). Insular cortex (INS) and inferior frontal gyrus (IFG) showed greater anticorrelation for the operator tactile attention (OTA) compared to operator auditory attention (OAA) group.

The whole brain analysis showed a positive correlation with the PCC time course in the angular gyrus (AG), medial frontal gyrus regions, and superior/inferior frontal gyrus, according to the well known topography of DMN (Figure 1). Furthermore, in both groups, a negative correlation with the PCC time course was observed in the inferior parietal lobe (IPL) and cingulate cortex. These regions overlap with the well known DAN nodes (Figure 1). Moreover bilateral insulae (INS) were found to be functionally anticorrelated with PCC. These areas showed a good spatial overlap with the spherical ROIs defined from the literature, as described in the method section. Figure 1 illustrates the connectivity values of these regions with PCC (mean z-fisher across subjects ± standard errors) for both groups during the experiment.

**Figure 2:**
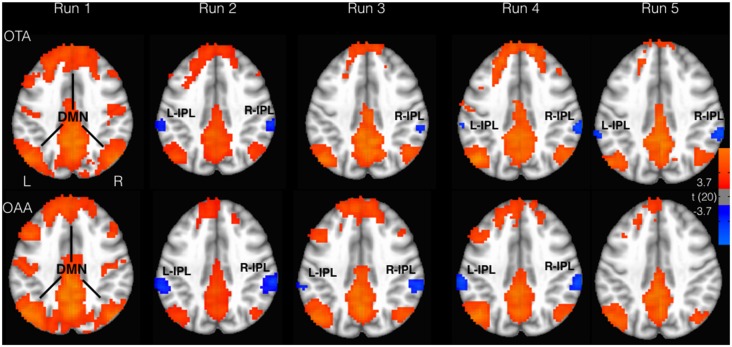
Areas correlated (red) and anticorrelated (blue) with PCC (*p* < 0.05, FDR corrected). Inferior parietal lobe (IPL) displayed greater anticorrelation for the OTA compared to OAA group. DMN, default mode network.

**Figure 3:**
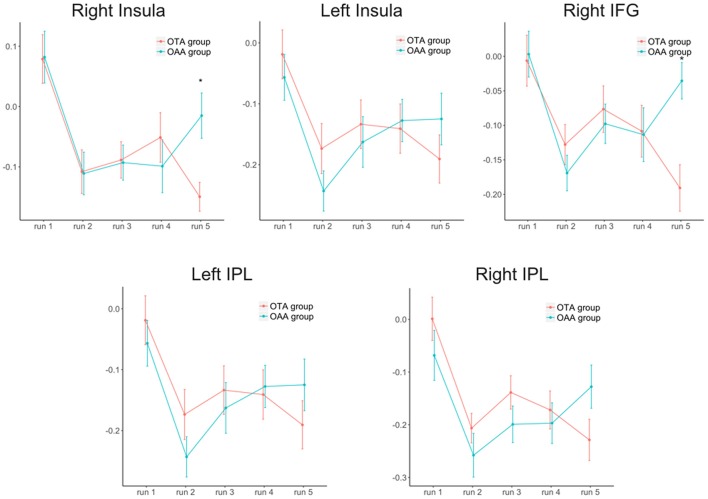
Trend of the anticorrelation (z-Fisher values ± SEM) over time for the two groups and the different regions of interest. IFG, inferior frontal gyrus; IPL, inferior parietal lobe. *Statistically significant values between groups after Bonferroni-Holm correction.

Considering the selected ROIs, the results of the MVM analyses performed on these values are shown in Table 1 and Figure 2. As illustrated in Figure 3, both groups revealed a significant increase of anticorrelation during touch as compared to respective baselines in the right, mid INS (main effect RUN, *F* = 10.74, *p* < 0.001), right inferior frontal gyrus (R-IFG; *F* = 6.85, *p* < 0.001), right (*F* = 11.21, *p* < 0.001) and left IPL (*F* = 7.33, *p* < 0.001). However, at run 5, i.e., after prolonged touch, these effects remained significant in the OTA group only.

The direct comparison between the two groups revealed a significant difference at run 5 for right mid insula (*t* = −3.02, *p* < 0.001), and right IFG (*t* = −3.63, *p* < 0.001), with the OTA group showing a larger anticorrelation with PCC as compared to the OAA group. A similar but non significant effect was observed in the L-IPL and R-IPL.

No statistically significant differences were found among the DMN nodes between conditions and across runs. Moreover, no correlation was observed between behavioral data and brain networks as well as brain patterns.

## Discussion

The current study aimed to explore the effect of sustained static touch on subjects brain functional connectivity while the operator is engaged in focused tactile/non-tactile attention tasks.

Results showed that prolonged sustained static touch applied by an operator engaged with focused tactile attention produced a significant increase of anticorrelation between PCC and right INS as well as right IFG but these functional connectivity changes are markedly different for the OTA and OAA conditions only after 15 min of touching. In other words, the present results showed that, if a particular cognitive status of the operator is sustained over time, it is able to elicit significant effects in the subjects’ functional connectivity between areas processing the interoceptive and attentional value of touch.

### Interoception and Touch

As far as the interoceptive aspect is concerned, the insula is known to be part of the interoceptive/salience neural network (Yarkoni et al., 2011); it integrates information from multiple brain regions, processing sensations ranging from physiologically driven motivational states to emotional awareness to somatosensory stimuli, including touch, which serves to maintain interoceptive homeostasis (Craig, 2002, 2009; Critchley et al., 2004; McGlone et al., 2014). Insula has reciprocal connections with the nPCC (Leech et al., 2012; Khalsa et al., 2014) exhibiting negative functional correlations mainly related to the allocation of task-positive or task-negative attentional resources based on interoceptive information (Fox et al., 2005; Uddin et al., 2009; Leech et al., 2012; Leech and Sharp, 2014).

Considering the insular effect during touch, it was demonstrated that the insular cortex is active in subjects receiving the touch—through a bottom-up process—(McGlone et al., 2014), with an insular somatotopic organization of CT-afferent fibers (McGlone et al., 2017). In addition, other research showed a top-down cognitive modulation of affective touch, demonstrating that subjects can cognitively modulate the response to the received touch during the “rubrich-rubthin” task (McCabe et al., 2008). These were further confirmed by a Rolls (2008) review that pointed out how different cognitive tasks, performed by subject, can modulate the effect of C-tactile afferent fibers. Interestingly, static touch seems to elicit similar but attenuate interoceptive effects in the insular cortex (Bolanowski et al., 2004; Ackerley et al., 2012). Notwithstanding these findings, studies considering the effects of the OTA status on the brain correlates of subjects receiving the touch are still lacking.

In this regard, our study showed that the anticorrelation between PCC, a central hub for the DMN and the insula, an important node of the salience network (SN; De Havas et al., 2012), is increased after prolonged static touch delivered by an operator engaged in a focused tactile attention task. Interestingly, our data also showed anticorrelation between PCC and left INS with a distinct pattern over time. Indeed, the PCC-left INS anticorrelation is shown to start and end earlier compared to that of PCC-right INS. It can be argued, therefore, that the negative correlation of the left INS could be a pre-mechanism aiming at tuning the subsequent re-representation of interoceptive information on the right INS (Craig, 2009).

### Touch and Attention

The current findings revealed an increased anticorrelation between the PCC and nodes of ACN, in particular the right IFG. Indeed, the right IFG was argued to come active with tasks (Liakakis et al., 2011), carried out by subjects receiving the touch, which demanded selective attention (Kemmotsu et al., 2005), in particular when performing an internal representation of movements (Iacoboni et al., 1999; Harrington et al., 2000), mainly associated to manual behavior (Aron et al., 2004; Matsubara et al., 2004).

Interestingly, previous studies demonstrated that switching between externally and internally oriented cognition is thought to be mediated via a competitive relationship between the DMN and the attentional control networks (ACN), DAN and SN (Fox et al., 2005; Fox and Raichle, 2007; Menon and Uddin, 2010). Therefore we might argue that using that type of attention task, an external operator can more efficiently modulate the switching between anticorrelated networks in subjects receiving the touch.

In addition, research supports a right-lateralized attention network (Sturm et al., 1999; Thiebaut de Schotten et al., 2011) with the insula playing a central role in the orientation of attention to behaviorally salient targets. Therefore, the effects induced by the operator might produce a modification of the salient afferent information in the subjects modifying the activity of attentional nodes.

However, the neuroimaging literature has pointed out also the role of the parietal cortex (Chambers et al., 2004; Macaluso and Driver, 2005), the somatosensory cortex (SI, SII and SIII; Burton and Sinclair, 2000; Sambo and Forster, 2011) and the cerebellar cortex (Burton et al., 2008) in the endogenous-oriented tactile spatial attention, whereas the right intraparietal sulcus (IPS), the (pre)motor cortices and caudate nucleus have been related to sustained tactile attention tasks (Sambo and Forster, 2011; Goltz et al., 2015), suggesting a wider attention network for tactile information processing, including frontal, parietal, occipital, cerebellar and limbic areas (Goltz et al., 2015).

### Further Considerations

In the literature, spontaneous anticorrelated activity was shown to be a consistent and organized phenomenon (Fox et al., 2005; Fransson, 2005; Hansen et al., 2015). Moreover, several studies demonstrated its relevance in different clinical conditions (Wang et al., 2007; Kelly et al., 2008; Keller et al., 2015; Yang et al., 2016) or in aging (Wu et al., 2011; Esposito et al., 2017), generally reporting a decrease of anticorrelation with pathology or aging.

It is interesting to point out that the touch protocol applied here is similar to that used in the context of manual therapy and touch-based interventions. Indeed, there are several osteopathic procedures that mimic the experimental study group where the operator is constantly touching the patient and contextually engaged into a focused tactile attention task, e.g., driving the attention towards the perception of myofascial movements (Tozzi et al., 2011; Pizzolorusso et al., 2014; Cerritelli et al., 2015; Ruffini et al., 2015; Tozzi, 2015).

In addition, the results on the insula might confirm previous conceptual article where D’Alessandro et al. (2016) hypothesized that manual therapy might exploit an interoceptive paradigm, arguing that the latter is an important component of the clinical effects of manual treatments speculating on the interoceptive role of the insula.

Therefore we might suggest that the current research, having provided initial lab-based evidence, would create the ground for further clinically-based studies.

In summary, the current study deepens our understanding of the brain mechanisms for touch processing. The results suggest a possible interoceptive role of the functional anticorrelation between right insula and PCC. While the right insula showed persistent and increasing anticorrelation during prolonged touch performed by an operator engaged into a tactile attention task, the left insula had a shorter response in both groups suggesting that this region might play a different role, e.g., in the initial activation of the interoceptive meaning of the touch. It is worth to mention here that the analysis was restricted to bivariate correlations between ROIs. Consequently, only changes of functional between-ROIs connectivity could be detected. It was not possible to determine how different tasks performed by the operator affected the activity in individual nodes, or how the nodes influenced one another across conditions. Techniques such as amplitude of spontaneous fluctuations analysis and spectral dynamic causal modeling could be used in the future to explore these questions.

Furthermore, although we pragmatically meant to reduce potential confounding sensory input between groups, we recognize the fact that the observed differences in brain connectivity are ultimately related to subtle differences in the physical properties of touch that are undetected by subjects but possibly act via subconscious sensory-based mechanisms.

Indeed, speculating on a plausible mechanism of action, we would argue that a touch focused on the myofascial movement would more accurately trigger the subject’s CT afferent fibers receptors (i.e., low-threshold mechanoreceptors), starting a cascade of bottom-up neurobiological events ending with distinct involvement of specific areas and networks in the brain. In addition, modifying the afferent input through this type of touch would change the tissue metabolic condition and thus its interoceptive inflow, possibly producing a central effect in terms of functional connectivity.

Note that an objective measurement of the physical properties of touching would require appropriate fMRI compatible equipment such as sensors placed on the subjects’ skin. Notwithstanding the finer physical features that would have been collected (i.e., not only the mean force applied during runs but also e.g., slight fluctuations of this force over time), this procedure would produce lack of skin-to-skin contact that was of primary importance for this experiment.

In conclusion, to the best of our knowledge, this is the first study in which the brain correlates of touch have been shown to be modulated by the cognitive task performed by the operator administering the touch. This is hypothesized to elicit interpersonal interactive processes including subconscious sensory-based mechanisms for the subjects and a particular attention status of the operator. Future studies should clarify the subtle physical features of touch originated by the different types of attention or cognitive tasks performed by the operator and producing the observed results.

## Author Contributions

FC, PC, FG and AF conceived the idea, drafted the first version of the article. AF and FG supervised the experiment, exported the data and reviewed the article. PC ran the statistical analysis, supervised the research and reviewed the article for intellectual content. All authors approved the final version.

## Conflict of Interest Statement

The authors declare that the research was conducted in the absence of any commercial or financial relationships that could be construed as a potential conflict of interest.

## Footnotes

^1^afni.nimh.nih.gov/afni

^2^http://surfer.nmr.mgh.harvard.edu

^3^http://cran.r-project.org/
